# Genomic predictions for economically important traits in Brazilian Braford and Hereford beef cattle using true and imputed genotypes

**DOI:** 10.1186/s12863-017-0475-9

**Published:** 2017-01-18

**Authors:** Mario L. Piccoli, Luiz F. Brito, José Braccini, Fernando F. Cardoso, Mehdi Sargolzaei, Flávio S. Schenkel

**Affiliations:** 1 Departamento de Zootecnia, Universidade Federal do Rio Grande do Sul, Porto Alegre, Brazil; 2 GenSys Consultores Associados S/S, Porto Alegre, Brazil; 3 Centre for Genetic Improvement of Livestock, University of Guelph, Guelph, Canada; 4 The Semex Alliance, Guelph, Canada; 5 Embrapa Pecuária Sul, Bagé, Brazil; 6 Conselho Nacional de Desenvolvimento Científico e Tecnológico, Brasília, Brazil

**Keywords:** Brazilian beef cattle, Genomic selection, Genotyping costs, Genomic breeding values, Imputation

## Abstract

**Background:**

Genomic selection (GS) has played an important role in cattle breeding programs. However, genotyping prices are still a challenge for implementation of GS in beef cattle and there is still a lack of information about the use of low-density Single Nucleotide Polymorphisms (SNP) chip panels for genomic predictions in breeds such as Brazilian Braford and Hereford. Therefore, this study investigated the effect of using imputed genotypes in the accuracy of genomic predictions for twenty economically important traits in Brazilian Braford and Hereford beef cattle. Various scenarios composed by different percentages of animals with imputed genotypes and different sizes of the training population were compared. De-regressed EBVs (estimated breeding values) were used as pseudo-phenotypes in a Genomic Best Linear Unbiased Prediction (GBLUP) model using two different mimicked panels derived from the 50 K (8 K and 15 K SNP panels), which were subsequently imputed to the 50 K panel. In addition, genomic prediction accuracies generated from a 777 K SNP (imputed from the 50 K SNP) were presented as another alternate scenario.

**Results:**

The accuracy of genomic breeding values averaged over the twenty traits ranged from 0.38 to 0.40 across the different scenarios. The average losses in expected genomic estimated breeding values (GEBV) accuracy (accuracy obtained from the inverse of the mixed model equations) relative to the true 50 K genotypes ranged from −0.0007 to −0.0012 and from −0.0002 to −0.0005 when using the 50 K imputed from the 8 K or 15 K, respectively. When using the imputed 777 K panel the average losses in expected GEBV accuracy was −0.0021. The average gain in expected EBVs accuracy by including genomic information when compared to simple BLUP was between 0.02 and 0.03 across scenarios and traits.

**Conclusions:**

The percentage of animals with imputed genotypes in the training population did not significantly influence the validation accuracy. However, the size of the training population played a major role in the accuracies of genomic predictions in this population. The losses in the expected accuracies of GEBV due to imputation of genotypes were lower when using the 50 K SNP chip panel imputed from the 15 K compared to the one imputed from the 8 K SNP chip panel.

**Electronic supplementary material:**

The online version of this article (doi:10.1186/s12863-017-0475-9) contains supplementary material, which is available to authorized users.

## Background

Brazil is a key player in the global beef market exporting throughout the whole world. Currently, Brazil has a herd of more than 212 million cattle [1], in which Zebu breeds are the most predominant in the national cattle population. However, there are other breeds with high economic impact in the Brazilian and international beef industry as well, such as Hereford and Braford (composite breed, which had genetic contribution from Zebu breeds in its development). Hereford and Braford breeds, together with Angus and Brangus account for 50% of the approximate eight million doses of beef cattle semen commercialized in Brazil in 2013 [2]. Much of this semen, as well as most live bulls sold are mated to Zebu females with the primary objective of improving carcass quality [3].

Genetic progress in Hereford and Braford breeding programs have been achieved through traditional genetic evaluations. However, incorporation of genomic information in livestock breeding programs (e.g. genomic selection, GS [4]) can result in higher and faster genetic progress [4–6], by decreasing generation interval, increasing accuracy of selection and facilitating incorporation of novel traits of economic importance in the current breeding programs [4, 7, 8]. GS has changed considerably the dairy cattle breeding systems, especially young bulls testing, where some countries have currently partially or completely eliminated the traditional progeny testing [9], with a subsequent reduction in costs in breeding programs [10].

The success of genomic predictions can be evaluated by accuracy of direct genomic breeding values (DGVs), which depends on many factors such as the level of linkage disequilibrium between markers and the quantitative trait loci (QTL), the number of animals in the training population, the heritability of the trait and the distribution of QTL effects over the genome [11]. Thus, the success of GS in dairy cattle, mainly in the Holstein breed, is associated with a large number of genotyped animals, a small effective population size (N_e_), large use of key sires world-wide and collaborations among countries for genotype sharing [11]. However, in the beef industry there are more challenges for the implementation of GS due to a larger effective population size compared to dairy cattle breeds, higher number of important beef cattle breeds world-wide, smaller number of key sires used across countries and also minimal collaboration among countries for genotyping sharing [9]. Some reported estimates of N_e_ for Holstein Friesian are 39 [12], 49 [13], 64 [14] and 90 [14]. For beef cattle some estimates of N_e_ are 234, 128, 185 and 303 for Angus, Devon, Hereford and Shorthorn, respectively [15], 207 and 285 for Angus and Charolais, respectively [16], and, 445 for American Red Angus [17].

There is a need to increase the size of training population for successful genomic predictions in beef cattle. However, genotyping costs in commercial herds is still a major constraint for implementation of GS. An alternative to reduce costs is to genotype individuals from commercial breeding programs with low-density single nucleotide polymorphisms (SNP) chip panels, which are more affordable for the producers. These low-density panels can then be imputed to a medium or high density SNP chip panel [18, 19] and used to predict genomic breeding values of the animals [20, 21]. It is important to investigate the impact of using imputed genotypes for genomic prediction of breeding values. Although the impact of using imputed genotypes for genomic prediction of breeding values has been investigated in different breeds [22, 23], this has not been reported in beef cattle breeds such as Brazilian Braford (Nellore x Hereford) and Hereford. Thus, the aim of this study was to investigate the accuracy of genomic predictions using true 50 K genotypes, as well as including alternative percentages of animals with imputed genotypes in the training population and different sizes of the training dataset in Brazilian Braford and Hereford beef cattle.

## Methods

### Genotypic and phenotypic data

Genotypic, phenotypic and pedigree datasets were obtained from the Conexão Delta G’s Genetic Improvement Program (Conexão Delta G, Dom Pedrito, Rio Grande do Sul, Brazil). The dataset contained approximately 520,000 animals from 97 farms located in the South, Southeast, Midwest and Northeast regions of Brazil. Out of these animals there were 683 Hereford and 2,997 Braford animals genotyped (born between 2008 and 2011) plus 130 sires (born between 1982 and 2010).

From the total of genotyped animals, there were 624 Hereford and 2,926 Braford animals genotyped with the Illumina BovineSNP50 panel (Illumina Inc.,San Diego, USA) and 59 Hereford and 71 Braford sires genotyped with the Illumina BovineHD panel (Illumina Inc., San Diego, USA). In addition, there were 88 Nellore bulls (Zebu breed used to develop Braford composite breed) from the Paint Breeding Program (Lagoa da Serra, Sertãozinho, São Paulo, Brazil) genotyped with the Illumina BovineHD panel.

### Genotype data editing

Single nucleotide polymorphisms that were not present in both 50 K and 777 K SNP chip panels were removed for imputation to the Illumina BovineSNP50 BeadChip (Illumina Inc., San Diego, CA). Missing genotypes (0.46%) in the 50 K SNP chip panel were previously imputed using FImpute software v.2.2 [24]. Only SNPs located on autosomes with GenCall score (≥0.15), call rate (≥0.90) and p-value for Hardy-Weinberg Equilibrium test (>10^−6^) were retained for further analyses. Quality control of individuals was based on GenCall score (≥0.15), call rate (≥0.90), heterozygosity deviation (limit of ± 3 SD), repeated sampling and paternity errors. The quality control for imputation to the 777 K SNP panel was the same as the one described before for the imputation to the 50 K SNP panel. The 8 K and 15 K SNP chip panels were used for imputation to 50 K SNP chip panel, and the 50 K SNP panel was used for imputation to the 777 K SNP panel [19] using FImpute v.2.2 [24]. A quality control as described before plus Minor Allele Frequency (MAF ≥ 0.05) was implemented for the genomic prediction of breeding values. Table 1 presents the number of individuals after the data quality control.

**Table 1:** Number of phenotypes, EBVs and genotypes for each economic trait in the training and validation population after data editing

Economic traits	Abbreviation	h^2b^	Phenotypes	Genotypes	Training population				Validation population			
					Progeny	Sire	Dam	Total	Progeny	Sire	Dam	Total
Traits included in the selection index												
Weight gain from birth to weaning (kg)	WGBW	0.25	354,255	3,305	2,231	91	3	2,325	944	9	27	980
Weight gain from weaning to yearling (kg)	WGWY	0.31	164,140	2,539	1,520	91	3	1,614	907	6	12	925
Conformation score at weaning (scores 1–5)	CW	0.25	348,020	2,970	1,896	91	3	1,990	944	9	27	980
Conformation score at yearling (scores 1–5)	CY	0.32	171,406	2,768	1,717	91	3	1,811	939	6	12	957
Precocity score at weaning (scores 1–5)	PW	0.25	330,312	2,961	1,887	91	3	1,981	944	9	27	980
Precocity score at yearling (scores 1–5)	PY	0.32	160,261	2,768	1,717	91	3	1,811	939	6	12	957
Muscularity score at weaning (scores 1–5)	MW	0.25	330,059	2,968	1,894	91	3	1,988	944	9	27	980
Muscularity score at yearling (scores 1–5)	MY	0.32	159,706	2,768	1,717	91	3	1,811	939	6	12	957
Scrotal circumference a (cm)	SCa^a^	0.43	46,823	1,581	623	85	0	708	865	5	3	873
Scrotal circumference aw (cm)	SCaw^a^	0.43	46,823	1,581	623	85	0	708	865	5	3	873
Traits not included in the selection index												
Birth weight (kg)	BW	0.33	221,038	3,434	2,401	88	3	2,492	905	10	27	942
Birth assistance score (scores 1–5)	BA	0.10	26,058	1,581	1,123	44	0	1,167	395	3	16	414
Size score at weaning (scores 1–5)	SW	0.25	140,681	2,911	1,848	81	3	1,932	944	8	27	979
Size score at yearling (scores 1–5)	SY	0.41	84,261	2,737	1,694	84	3	1,781	939	5	12	956
Prepuce (navel) score at weaning (scores 1–5)	NW	0.46	265,802	3,343	2,291	89	2	2,382	927	7	27	961
Prepuce (navel) score at yearling (scores 1–5)	NY	0.41	122,409	2,825	1,793	89	2	1,884	924	5	12	941
Hair length score at weaning (scores 1–3)	HW	0.23	110,163	1,579	612	86	2	700	846	6	27	879
Hair length score at yearling (scores 1–3)	HY	0.31	73,621	2,480	1,561	88	2	1,651	813	4	12	829
Ticks resistance (ticks unit)	TR	0.19	12,099	1,640	594	60	0	654	948	3	3	954
Ocular pigmentation score (scores 1–3)	OP	0.20	139,082	1,635	587	90	1	678	921	9	27	957

^a^ SCa is the scrotal circumference adjusted for age at yearling and SCaw is the scrotal circumference adjusted for age and weight at yearling; ^b^ The heritability estimates were obtained prior to this study using the DMU package [49]

### Traits

Conexão Delta G’s Genetic Improvement Program - Hereford and Braford (Nellore x Hereford) started around 1970. During its early stages animals were selected based on a selection index that included weight gain, scrotal circumference and conformation score traits [25]. In 1975, other traits such as precocity, muscularity and body size scores [26] were incorporated into the selection index. In the 1990, body size score was excluded from the selection index. Furthermore, there are other traits not included in the selection index, which are used for culling of animals as well. Therefore, the traits included in this study can be divided in two groups: 1) traits that make up the selection index used by Conexão Delta G’s Genetic Improvement Program; and 2) traits that are not included in the selection index, but are used for independent culling selection. The current selection index is based on 25% for weight gain from birth to weaning (WGBW), 25% for weight gain from weaning to yearling (WGWY), 4% for conformation score at weaning (CW), 4% for conformation score at yearling (CY), 8% for precocity score at weaning (PW), 8% for precocity score at yearling (PY), 8% for muscularity score at weaning (MW), 8% for muscularity score at yearling (MY), 5% for scrotal circumference adjusted for age at yearling (SCa) and 5% for scrotal circumference adjusted for age and weight at yearling (SCaw). On the other hand, the traits that are not included in the selection index are: birth weight (kg, BW), birth assistance score (scores 1–5, BA), size score at weaning (scores 1–5, SW), size score at yearling (scores 1–5, SY), prepuce (navel) score at weaning (scores 1–5, NW), prepuce (navel) score at yearling (scores 1–5, NY), hair length score at weaning (scores 1–3, HW), hair length score at yearling (scores 1–3, HY), ticks resistance (ticks unit, TR) and ocular pigmentation score (scores 1–3, OP).

The independent culling level was carried out systematically since the beginning of the Conexão Delta G’s Genetic Improvement Program for BW, BA and OP traits, particularly in Hereford, and NW, NY, HW and HY in Braford. The SW and SY traits were part of the selection index between the 1970s and 1990s while the selection of TR has been performed with greater emphasis on young bulls in the last decade.

### Traditional genetic evaluation

The package used to obtain the estimated breeding values (EBVs) for each trait was written in Fortran language and developed by GenSys (GenSys Consultores Associados, Porto Alegre, Brazil). Contemporary group was defined based on farm, year-season, sex, and management group. The traits WGBW, CW, CY, PW, PY, MW, MY and SW were pre-adjusted for dam age, birth date, breed, dominance and epistasis effects and environmental interactions (latitude). WGWY was pre-adjusted for calf age, while CW, CY, PW, PY, MW, MY and SY were pre-adjusted for calf and dam age. SCa was adjusted for age at yearling and SCaw was adjusted for age and weight at yearling. TR was pre-adjusted for additive effects of breed. A connectedness analysis was performed prior to each genetic evaluation. The degree of connectedness among contemporary groups was measured based on genetic connections of animals and its common relatives. Genetic connections were weighted by the degree of additive relationship between animals [27, 28]. To be considered connected, contemporary groups were defined as a minimum of 10 genetic direct connections. All individuals not assigned to a contemporary group were excluded from the genetic evaluations.

The general model used for the genetic evaluations was: y_ijkl_ = μ + cg_i_ + a_j_ + m_k_ + pe_k_ + e_ijkl_, where *y*_*ijkl*_ is the phenotype for the animal *l*, pre-adjusted for the known environmental effects (individual age, dam age and birth date) and genetic fixed effects (breed, dominance, epistatic, complementary and interactions with latitude); *μ* is the general mean for the trait; c*g*_*i*_ is the effect of contemporary group *i* (fixed effect); *a*_*j*_ is the genetic direct effect of animal *j* (random effect); *m*_*k*_ is the maternal genetic effect of cow *k* (random effect); *pe*_*k*_ is the permanent environment effect due to the cow *k* (random effect); and *e*_*ijkl*_ is the residual effect associated to the observation *ijkl*. The required variance components were estimated using Restricted Maximum Likelihood (REML). A robust estimation procedure regarding to the heterogeneity of residual variance within contemporary groups [29] was used. The robust estimation procedure allows observations from cg with large residual variance to have reduced influence, while not giving to much weight to observation from cg with low residual variance [29].

Two EBV sets were generated: the first one was estimated using all available information to date while the second set was estimated using information from all animals born before 2010. These two sets of EBVs were then used as pseudo-phenotypes in the genomic prediction models for validation and training populations, respectively.

### De-regressed EBVs

The second set of EBVs (for the training population) was de-regressed and used as pseudo-phenotypes to estimate genomic markers effects. The approach described by VanRaden and Wiggans [30] was used to calculate de-regressed EBVs using EBVs and reliabilities of genotyped animals and their sires and dams. De-regressed EBVs were calculated for animals of the training population with EBV reliability greater than the overall mean (*r* ^2^ = 0.09) and that satisfied the following condition: $abs\left(\frac{\left(EBV- dEBV\right)}{sdEBV}\right)\le 10 sdEBV$, where *abs* represents the absolute value, *EBV* is the estimated breeding value, *dEBV* is the de-regressed EBV and *sdEBV* is the standard deviation of the EBVs.

### Prediction of DGV and GEBV

Direct genomic values (DGVs) were estimated using GBLUP method as described in VanRaden [7] for all the twenty traits (Table 1), using either 50 K or 777 K SNP chip panels and de-regressed EBVs. The GEBV software was used for the analysis [31]. The following linear model was implemented: **y** **= 1**_**n**_ **μ** **+** **Zg** **+** **e**, where *y* is the vector of de-regressed EBV for the trait, *μ* is the overall mean, *1*_*n*_ is a vector of ones, ***Z*** is the design matrix that relates de-regressed EBVs to animals, ***g*** is the vector of DGV to be predicted, and ***e*** is the vector of residual effects. It was assumed that **g** ~ N (0, G*σ^2^_g_) where σ^2^_g_ is the additive genetics variance and G* is a combined relationship matrix (80% genomic relationship and 20% pedigree-based relationship), and **e ~** N (0**, R**σ^2^_e_) where σ^2^_e_ is the residual variance and **R** is a diagonal matrix whose elements account for the differences in reliabilities of the de-regressed EBVs. The reason for using a combined relationship matrix is due to the fact that previous studies, also performed in Brazil, reported gains in accuracies when adding 20% pedigree-based relationship (e.g. [32]).

The genomic estimated breeding values (GEBV) were estimated using the blending procedure outlined by Hayes et al. [11] and described as: $\mathbf{GEBV}=\frac{{\mathbf{r}}_{\mathbf{DGV}}^{\mathbf{2}}*\mathbf{D}\mathbf{G}\mathbf{V}+{\mathbf{r}}_{\mathbf{EBV}}^{\mathbf{2}}*\mathbf{E}\mathbf{B}\mathbf{V}}{{\mathbf{r}}_{\mathbf{DGV}}^{\mathbf{2}}+{\mathbf{r}}_{\mathbf{EBV}}^{\mathbf{2}}}$, where **r**_**DGV**_^2^ and **r**_**EBV**_^2^ are the reliability of DGV and EBV, respectively.

### Training and validation populations

For the genomic predictions as described in the previous section, the dataset was split into two groups: training and validation populations. To simulate what would happen in practice (genotype and phenotypes from older animals used to predict breeding values of younger animals), the training population included all animals born before 2011, while the validation group included all the animals born in 2011 (youngest animals). Training and validation groups varied in size for each trait (Table 1). The training group had 100% of true genotypes or alternatively, between 10% and 60% (10%, 20%, …, 60%) of imputed genotypes in the first two groups of scenarios. In the third group of scenarios, the training group, had 91% of imputed genotypes because only 212 animals were genotyped with 777 K chip panel. In the validation groups, only true genotypes were included.

### Genomic prediction scenarios

Three groups of genomic prediction scenarios were designed to mimic situations where different proportion of animals with imputed genotypes (derived from alternate low-/medium- density SNP chip panels) were included in the training set. The first two groups of scenarios were created based on animals genotyped (mimicked from 50 K SNP chip panel) with 8 K and 15 K SNP chip panels and imputed to the 50 K SNP chip panel [19]. For this study, the two best scenarios based on concordance rate and allelic R^2^ were used: 8 K scenario (concordance rate: 0.952 and allelic R^2^: 0,927) and 15 K scenario (concordance rate: 0.973 and allelic R^2^: 0,962). The 20 K panel was slightly superior to the 8 K panel. However, various markers from the 20 K are not included in the 50 K and 777 K, and therefore, when matching panels for imputation 8 K and 20 K become very similar [19].

The first group of scenarios (SCE1) was created with different percentages of animals with imputed genotypes and unequal training population sizes (separated based on birth year, as described in the previous section). The second group of scenarios (SCE2) was also created with different percentages of animals with imputed genotypes, however, with same size training populations. The third group of scenarios (SCE3) was based on animals genotyped using the 50 K SNP chip panel and imputed to the 777 K SNP chip panel. More details for all scenarios are shown in the Table 2.

**Table 2:** Number of animals with true and imputed genotypes in each scenario in the training set for the weight gain from birth to weaning (WGBW) trait^a^

Scenarios SCE1 – 50 K SNP panel				Scenarios SCE2 – 50 K SNP panel				Scenario SCE3 – 777 K SNP panel			
Level^b^	True	Imputed genotypes	Total	Level^b^	True	Imputed genotypes	Total	Level^b^	True	Imputed genotypes	Total
0	2,325	0	2,325	0	2.325	0	2,325	91	212	2,113	2,325
10	930	103	1,033	10	2,092	233	2,325				
20	930	233	1,163	20	1,860	465	2,325				
30	930	399	1,329	30	1,627	698	2,325				
40	930	620	1,550	40	1,395	930	2,325				
50	930	930	1,860	50	1,162	1,163	2,325				
60	930	1,395	2,325	60	930	1,395	2,325				

^a^ The same criteria were used to define the scenarios of other traits, but the number of genotypes varies for each trait; ^b^ Percentage of animals with imputed genotypes used in the training population

### Comparisons between scenarios

The prediction accuracies of GEBV were used to compare the scenarios evaluated. The prediction accuracies were calculated as Pearson’s correlation between DGVs and EBVs (validation accuracy) from the validation population. Accuracy obtained from the mixed model equations (expected accuracy) in the validation population was used to quantify losses in GEBV accuracy due to the use of imputed genotypes compared to the true 50 K SNP chip panel. Expected accuracy was also used to quantify the gain in breeding value accuracies when using molecular marker information in the EBV estimation. The factors affecting validation accuracies and losses in expected GEBV accuracies were tested by carrying out an analysis of variance in the ANOVA procedure of SAS version 9.2 (SAS Inst. Inc., Cary, NC).

## Results

### Phenotypic and genotypic data

As shown in Table 1, the average heritability estimates (± SD) for the traits included in this study was 0.30 ± 0.09 and it ranged from 0.10 (BA) to 0.46 (NW). The average number of individuals (± SD) in the training population was 1,603.4 ± 594.7 and ranged from 654 (TR) to 2,492 (BW). The average number of individuals in the validation population (± SD) was 913.7 ± 122.1 and ranged from 414 (BA) to 980 (WGBW, CW, PW and MW).

### Adding alternative imputed genotypes to increase the size of training population (SCE1)

In the SCE1, we investigated the prediction accuracies of genomic breeding values when increasing the size of the training population by inclusion of imputed genotypes (imputed from 8 K or 15 K to 50 K). Tables 1 and 2 show the number of animals in the training and validation population within scenario evaluated and total number of genotypes (true and imputed). The average validation accuracies for the traits included in the selection index ranged from 0.29 to 0.31 (Table 3), while for the traits not included in the selection index, it ranged from 0.47 to 0.49 (Table 4). However, for the traits related to fitness (NW, NY, HW, HY, TR and OP), the average validation accuracy ranged from 0.63 to 0.65 (Table 4). As shown in Table 5, higher accuracies were observed for the majority of the traits when including a higher proportion of imputed genotypes in the training population.

**Table 3:** DGVs validation accuracies for the SCE1 scenarios and traits included in the selection index^a, b^

Traits^c^	8 K^d^						15 K^d^						50 K^d^					
	10^e^	20^e^	30^e^	40^e^	50^e^	60^e^	10^e^	20^e^	30^e^	40^e^	50^e^	60^e^	10^f^	20^f^	30^f^	40^f^	50^f^	60^f^
WGBW	0.32	0.33	0.34	0.34	0.34	0.32	0.32	0.33	0.34	0.33	0.33	0.32	0.31	0.33	0.34	0.33	0.34	0.32
WGWY	0.33	0.33	0.33	0.34	0.36	0.35	0.33	0.33	0.33	0.33	0.36	0.35	0.33	0.33	0.33	0.33	0.36	0.35
CW	0.24	0.27	0.29	0.30	0.31	0.29	0.25	0.28	0.29	0.30	0.31	0.28	0.25	0.28	0.29	0.30	0.31	0.29
CY	0.29	0.29	0.29	0.28	0.29	0.26	0.29	0.29	0.29	0.29	0.29	0.26	0.29	0.29	0.29	0.29	0.29	0.26
PW	0.28	0.29	0.30	0.31	0.33	0.31	0.27	0.29	0.29	0.31	0.33	0.31	0.27	0.29	0.30	0.31	0.33	0.31
PY	0.32	0.32	0.31	0.32	0.35	0.33	0.32	0.32	0.31	0.32	0.35	0.34	0.32	0.32	0.31	0.33	0.35	0.34
MW	0.32	0.33	0.34	0.34	0.37	0.35	0.32	0.33	0.34	0.34	0.37	0.35	0.31	0.33	0.34	0.34	0.37	0.35
MY	0.35	0.35	0.33	0.34	0.37	0.34	0.35	0.35	0.33	0.35	0.38	0.35	0.35	0.35	0.33	0.34	0.38	0.35
SCa	0.21	0.21	0.20	0.20	0.17	0.18	0.22	0.23	0.21	0.21	0.17	0.18	0.22	0.22	0.21	0.20	0.18	0.18
SCaw	0.24	0.23	0.22	0.21	0.17	0.20	0.25	0.24	0.22	0.22	0.18	0.19	0.25	0.25	0.23	0.22	0.18	0.20
Average	0.29	0.30	0.30	0.30	0.31	0.29	0.29	0.30	0.30	0.30	0.31	0.29	0.29	0.30	0.30	0.30	0.31	0.30

^a^ DGV validation accuracy means Pearson’s correlation between DGVs and EBVs in the validation population; ^b^ SCE1 scenario that the number of animals and the percentage of animals with imputed genotypes in the training population varied; ^c^Trait abbreviations are presented in Table 1; ^d^ 8 K: means that the base panel is the 8 K SNP panel imputed to the 50 K SNP panel; 15 K: means that the base panel is the 15 K SNP panel imputed to the 50 K SNP panel; 50 K: means the true 50 K SNP panel; ^e^ Percentage of animals with imputed genotypes in the training population; ^f^ The percentage 10 to 60 correspond to the similar analysis carried out with the 8 K and 15 K SNP panels, but using only true genotypes

**Table 4:** DGVs validation accuracies for the SCE1 scenarios and traits included not included in the selection index^a, b^

Traits^c^	8 K^d^						15 K^d^						50 K^d^					
	10^e^	20^e^	30^e^	40^e^	50^e^	60^e^	10^e^	20^e^	30^e^	40^e^	50^e^	60^e^	10^f^	20^f^	30^f^	40^f^	50^f^	60^f^
BW	0.21	0.21	0.22	0.21	0.21	0.22	0.21	0.21	0.23	0.21	0.21	0.21	0.20	0.21	0.22	0.20	0.20	0.21
BA	0.12	0.14	0.12	0.13	0.14	0.14	0.14	0.15	0.14	0.14	0.15	0.15	0.13	0.14	0.13	0.13	0.14	0.14
SW	0.28	0.30	0.31	0.32	0.31	0.33	0.28	0.30	0.31	0.31	0.31	0.33	0.27	0.30	0.31	0.31	0.31	0.33
SY	0.29	0.30	0.30	0.30	0.33	0.36	0.30	0.31	0.31	0.30	0.33	0.35	0.30	0.31	0.31	0.30	0.33	0.36
NW	0.47	0.49	0.49	0.50	0.51	0.53	0.47	0.48	0.49	0.50	0.51	0.53	0.47	0.49	0.49	0.50	0.52	0.54
NY	0.46	0.48	0.50	0.50	0.52	0.54	0.46	0.48	0.50	0.50	0.51	0.54	0.46	0.48	0.50	0.50	0.52	0.54
HW	0.73	0.72	0.72	0.71	0.71	0.71	0.73	0.73	0.72	0.71	0.71	0.71	0.73	0.73	0.72	0.71	0.72	0.71
HY	0.79	0.79	0.78	0.78	0.81	0.81	0.79	0.79	0.78	0.79	0.81	0.81	0.80	0.79	0.79	0.79	0.81	0.81
TR	0.65	0.64	0.63	0.62	0.62	0.62	0.65	0.64	0.63	0.62	0.62	0.62	0.65	0.64	0.63	0.62	0.62	0.62
OP	0.70	0.69	0.69	0.67	0.67	0.66	0.70	0.69	0.68	0.67	0.66	0.66	0.70	0.69	0.69	0.68	0.67	0.66
Average	0.47	0.48	0.48	0.47	0.48	0.49	0.47	0.48	0.48	0.48	0.48	0.49	0.47	0.48	0.48	0.47	0.48	0.49
Overall mean	0.38	0.39	0.39	0.39	0.39	0.39	0.38	0.39	0.39	0.39	0.39	0.39	0.38	0.39	0.39	0.39	0.40	0.39

^a^ DGV validation accuracy means Pearson’s correlation between DGVs and EBVs in the validation population; ^b^ SCE1 scenario that the number of animals and the percentage of animals with imputed genotypes in the training population varied; ^c^Trait abbreviations are presented in Table 1; ^d^ 8 K: means that the base panel is the 8 K SNP panel imputed to the 50 K SNP panel; 15 K: means that the base panel is the 15 K SNP panel imputed to the 50 K SNP panel; 50 K: means the true 50 K SNP panel; ^e^ Percentage of animals with imputed genotypes in the training population; ^f^ The percentage 10 to 60 correspond to the similar analysis carried out with the 8 K and 15 K SNP panels, but using only true genotypes

**Table 5:** Results of analysis of variance of the DGV validation accuracies for the SCE1 scenarios^a, b, c^

Trait	Panel^d^			Scenario^e^				
	8–15	8–50	15–50	10–60	20–60	30–60	40–60	50–60
WGBW	* (8)	ns	ns	* (60)	* (20)	* (30)	* (40)	* (50)
WGWY	ns	ns	ns	* (60)	* (60)	* (60)	* (60)	ns
CW	ns	* (50)	ns	* (60)	* (60)	ns	* (40)	* (50)
CY	* (15)	* (50)	ns	* (10)	* (20)	* (30)	* (40)	* (50)
PW	ns	ns	ns	* (60)	* (60)	* (60)	ns	* (50)
PY	ns	ns	ns	* (60)	* (60)	* (60)	* (60)	* (50)
MW	ns	ns	* (15)	* (60)	* (60)	* (60)	* (60)	* (50)
MY	ns	ns	ns	ns	ns	* (60)	ns	* (50)
SCa	* (15)	ns	ns	* (10)	* (20)	* (30)	* (40)	ns
SCaw	* (15)	* (50)	* (50)	* (10)	* (20)	* (30)	* (40)	* (60)
BW	ns	* (8)	* (15)	* (60)	ns	* (30)	* (60)	* (60)
BA	* (15)	ns	* (15)	* (60)	ns	* (60)	* (60)	ns
SW	* (8)	* (8)	ns	* (60)	* (60)	* (60)	* (60)	* (60)
SY	ns	ns	ns	* (60)	* (60)	* (60)	* (60)	* (60)
NW	ns	ns	ns	* (60)	* (60)	* (60)	* (60)	* (60)
NY	ns	ns	ns	* (60)	* (60)	* (60)	* (60)	* (60)
HW	* (15)	* (50)	* (50)	* (10)	* (20)	* (30)	ns	ns
HY	* (15)	* (50)	* (50)	* (60)	* (60)	* (60)	* (60)	ns
TR	ns	* (50)	* (50)	* (10)	* (20)	* (30)	ns	* (60)
OP	* (15)	* (50)	* (50)	* (10)	* (20)	* (30)	* (40)	* (50)

^a^ DGV validation accuracy means Pearson’s correlation between DGVs and EBVs in the validation population; ^b^ SCE1 scenario that the number of animals and the percentage of animals with imputed genotypes in the training population varied; ^c^ “*” means that there was a significant difference (*P* < 0.05). The value between brackets indicates which panel/scenario had higher estimated accuracy. “ns” means that there was no significant difference (*P* > 0.05); ^d^ 8,15 and 50 means the 8 K, 15 K and 50 K SNP panel. 8–15, 8–50 and 15–50 are the contrast between the two panels; ^e^ 10,20,30,40,50 and 60 means the percentage of imputed genotypes. 10–60, 20–60, 30–60, 40–60, 50–60 are the contrasts between the two percentages of the imputed genotypes

The differences between the 8 K and 15 K SNP panels were not significant (*P* > 0.05) for the majority of the traits (55%) and in general, when there was a significant difference, 15 K performed better than 8 K (Table 5). When comparing 8 K and 15 K to the true 50 K SNP, significant differences (*P* < 0.05) were observed in 45% and 40% of the traits, respectively. In general, the accuracies were higher when using the true 50 K SNP panel. However, for BW higher accuracies were observed when using imputed genotypes from 8 K and 15 K.

When evaluating the size and percentage of animals with imputed genotypes in the training population in SCE1 scenarios (Table 5), 86% of the comparisons were statistically significant (*P* < 0.05). In general, larger training populations (i.e. including larger proportion of imputed genotypes) provided greater prediction accuracies. Regarding to the panel used, 60% of the comparisons between 8 K and 15 K SNP chip panels and the true 50 K panel were statistically significant (*P* < 0.05) and in most cases the true 50 K SNP panel provided greater accuracies (Table 5). For the 8 K and 15 K SNP panel the average losses in expected GEBV accuracy were between −0.004 and −0.0011. More details about the losses in GEBV accuracies are presented in Additional file 1.

### Comparing different proportion of imputed genotypes keeping constant the size of the training population (SCE2)

Table 6 shows the accuracies of genomic predictions in the SCE2 scenarios, where the training population size was held constant but the percentage of imputed animals varied from 0% to 60%. The average accuracies ranged between 0.29 and 0.30 for the traits included in the selection index and for the traits not included in the selection index they were all the same (0.49). There were no significant differences among the alternate percentage of imputed animals (*P* > 0.05) for all traits (Table 7). The comparison of the 8 K and 15 K SNP chip panels to the true 50 K SNP panel showed significant differences (*P* < 0.05) in 45% and 60% of the cases, respectively (Table 7). When there were significant differences, in general, the true 50 K SNP panel performed better than the imputed genotypes. The differences between the 8 K and 15 K SNP panels were not significant (*P* > 0.05) for the majority of traits (Table 7).

**Table 6:** DGV validation accuracy in the validation population for the SCE2 and SCE3 scenarios^a, b^

Traits^c^	8 K^d^						15 K^d^						50 K^d^	777 K^d, e^
	10	20	30	40	50	60	10	20	30	40	50	60		
Traits in the selection index														
WGBW	0.33	0.33	0.32	0.33	0.32	0.32	0.32	0.32	0.32	0.32	0.32	0.32	0.32	0.34
WGWY	0.35	0.35	0.35	0.35	0.35	0.35	0.35	0.35	0.35	0.35	0.35	0.35	0.35	0.36
CW	0.29	0.29	0.29	0.29	0.29	0.29	0.28	0.28	0.28	0.28	0.29	0.29	0.29	0.32
CY	0.27	0.26	0.26	0.26	0.25	0.26	0.26	0.26	0.26	0.26	0.26	0.26	0.26	0.29
PW	0.31	0.31	0.31	0.31	0.31	0.32	0.31	0.30	0.30	0.31	0.31	0.31	0.31	0.32
PY	0.34	0.34	0.34	0.33	0.33	0.34	0.34	0.34	0.34	0.34	0.33	0.34	0.34	0.34
MW	0.35	0.35	0.35	0.35	0.35	0.35	0.35	0.35	0.35	0.35	0.35	0.35	0.35	0.37
MY	0.35	0.35	0.35	0.34	0.34	0.35	0.35	0.35	0.34	0.34	0.34	0.35	0.35	0.36
SCa	0.18	0.19	0.18	0.19	0.19	0.18	0.18	0.19	0.19	0.19	0.19	0.19	0.18	0.17
SCaw	0.20	0.20	0.20	0.20	0.20	0.19	0.20	0.20	0.20	0.20	0.20	0.20	0.20	0.20
Average	0.30	0.30	0.30	0.30	0.29	0.30	0.29	0.29	0.29	0.29	0.29	0.30	0.30	0.31
Traits not in the selection index														
BW	0.22	0.21	0.21	0.22	0.21	0.21	0.21	0.21	0.21	0.21	0.21	0.21	0.21	0.21
BA	0.13	0.14	0.14	0.14	0.14	0.15	0.15	0.15	0.15	0.15	0.15	0.16	0.14	0.11
SW	0.33	0.34	0.34	0.34	0.33	0.33	0.33	0.33	0.33	0.33	0.33	0.33	0.33	0.36
SY	0.36	0.36	0.36	0.35	0.35	0.35	0.36	0.36	0.36	0.35	0.36	0.36	0.36	0.37
NW	0.54	0.54	0.53	0.53	0.53	0.53	0.54	0.54	0.54	0.54	0.54	0.54	0.54	0.56
NY	0.54	0.54	0.54	0.54	0.54	0.54	0.54	0.54	0.54	0.55	0.55	0.54	0.54	0.57
HW	0.71	0.71	0.71	0.71	0.71	0.71	0.71	0.71	0.71	0.71	0.71	0.71	0.71	0.71
HY	0.81	0.81	0.81	0.81	0.81	0.81	0.81	0.81	0.81	0.80	0.81	0.81	0.81	0.80
TR	0.62	0.62	0.62	0.62	0.62	0.62	0.62	0.62	0.62	0.62	0.62	0.62	0.62	0.62
OP	0.66	0.66	0.66	0.66	0.66	0.66	0.66	0.66	0.66	0.66	0.66	0.66	0.66	0.64
Average	0.49	0.49	0.49	0.49	0.49	0.49	0.49	0.49	0.49	0.49	0.49	0.49	0.49	0.50
Overall mean	0.39	0.39	0.39	0.39	0.39	0.39	0.39	0.39	0.39	0.39	0.39	0.39	0.39	0.40

^a^ DGV validation accuracy means Pearson’s correlation between DGVs and EBVs in the validation population; ^b^ SCE2 scenario that the percentage of animals with imputed genotypes in the training population varied and SCE3 scenario was created with only one percentage of animals with imputed genotypes and only one training population size; ^c^Abreviation descriptions are presented in Table 1; ^d^ 8 K: means that the base panel is the 8 K SNP panel imputed to the 50 K SNP panel; 15 K: means that the base panel is the 15 K SNP panel imputed to the 50 K SNP panel; 50 K: means the true 50 K SNP panel; 777 K: means that the base panel is the 50 K SNP panel imputed to the 777 K SNP panel; ^e^ When the 777 K SNP panel was used, all animals had their genotypes imputed from the 50 K SNP panel to the 777 K SNP panel, except for 212 animals in the training population (SCE3)

**Table 7:** Results of analysis of variance of the DGV validation accuracies for the SCE2 scenarios^a,b,c^

Trait	Panel^d^			Scenario^e^				
	8–15	8–50	15–50	10–60	20–60	30–60	40–60	50–60
WGBW	ns	ns	ns	ns	ns	ns	ns	ns
WGWY	ns	* (50)	* (50)	ns	ns	ns	ns	ns
CW	* (8)	ns	* (50)	ns	ns	ns	ns	ns
CY	ns	ns	* (50)	ns	ns	ns	ns	ns
PW	ns	* (50)	* (50)	ns	ns	ns	ns	ns
PY	ns	* (50)	* (50)	ns	ns	ns	ns	ns
MW	ns	ns	* (50)	ns	ns	ns	ns	ns
MY	* (8)	ns	* (50)	ns	ns	ns	ns	ns
SCa^f^	ns	* (8)	* (15)	ns	ns	ns	ns	ns
SCaw^f^	* (15)	* (50)	ns	ns	ns	ns	ns	ns
BW	ns	ns	ns	ns	ns	ns	ns	ns
BA	* (15)	ns	* (15)	ns	ns	ns	ns	ns
SW	* (8)	* (8)	ns	ns	ns	ns	ns	ns
SY	ns	ns	ns	ns	ns	ns	ns	ns
NW	* (15)	ns	ns	ns	ns	ns	ns	ns
NY	* (15)	* (50)	ns	ns	ns	ns	ns	ns
HW	* (15)	* (50)	* (50)	ns	ns	ns	ns	ns
HY	ns	ns	ns	ns	ns	ns	ns	ns
TR	* (8)	* (8)	* (50)	ns	ns	ns	ns	ns
OP	ns	ns	* (50)	ns	ns	ns	ns	ns

^a^ DGV validation accuracy means Pearson’s correlation between DGVs and EBVs in the validation population; ^b^ SCE2 scenario that the percentage of animals with imputed genotypes in the training population varied; ^c^ “*” means that there was a significant difference (*P* < 0.05). The value between brackets indicates which panel/scenario had higher estimated accuracy. “ns” means that there was no significant difference (*P* > 0.05); ^d^ 8,15 and 50 means the 8 K, 15 K and 50 K SNP panel. 8–15, 8–50 and 15–50 are the contrasts between the two panels; ^e^ 10, 20, 30, 40, 50 and 60 means the percentage of imputed genotypes. 10–60, 20–60, 30–60, 40–60, 50–60 is the contrast between the two percentages of the imputed genotypes; ^f^ SCa is the scrotal circumference adjusted for age at yearling and SCaw is the scrotal circumference adjusted for age and weight at yearling

Losses in expected GEBV accuracy were measured within each level of the scenario in relation to the same level of the scenario using only the actual genotypes. All losses were statistically different from actual (not imputed) 50 K SNP panel (*P* < 0.05) and were higher when using the 8 K SNP panel in relation to the 15 K SNP panel. For the 8 K and 15 K SNP panel the average losses in expected GEBV accuracy were between −0.0002 and −0.0011 across scenarios (Additional file 2). In general, a higher proportion of imputed genotypes in the training population in SCE2 was associated with larger reductions in accuracies.

There were no statistically significant differences (*P* > 0.05) in validation accuracies when including different percentage of imputed animals (Table 7). When comparing the SNP chip panels, there were no significant differences for 60% of the traits (*P* > 0.05) between the 8 K and 15 K SNP chip panels and the true 50 K SNP chip panel (Table 7). The losses in GEBV accuracies are presented in Additional file 2. In brief, losses in expected GEBV accuracy were statistically different from true 50 K genotypes (*P* < 0.05) for traits not included in the selection index. Using imputed genotypes from 8 K and 15 K to the 50 K SNP chip panel, the average losses in GEBV expected accuracy ranged between −0.0004 and −0.0013 and −0.0003 to −0.0013, respectively.

### Comparing prediction accuracy of genomic breeding values using 50 K or 777 K SNP chips (SCE3)

We also investigated the use of a 777 K SNP chip panel imputed from 50 K (SCE3). The average DGV validation accuracies were 0.31 and 0.50 for traits included or not in the selection index, respectively (Table 6). The average loss in expected GEBV accuracy was −0.0021 (Additional file 2).

### Including genomic information

The average expected EBV accuracy in the training population was 0.64 and ranged from 0.52 to 0.74. For the validation population, the average expected EBV accuracy was 0.63 and ranged between 0.51 and 0.73. Average expected GEBV accuracy was 0.66 for the scenario with all animals and 60% imputed genotypes (SCE1-60% and SCE2-60%) and 0.65 for the SCE3 scenario. The increase in average expected GEBV accuracy in the validation population by adding the information of the markers was 0.03. The average expected DGV accuracy across traits was 0.40 (Table 8). The increase in expected GEBV accuracy by adding marker information was about 0.02 in all scenarios.

**Table 8:** Expected EBV accuracy in the training and validation population and expected GEBV and DGV accuracy in the validation population in the scenario with the largest training population^a^

Trait^c^	EBV training	EBV validation	8 K^b^		15 K^b^		50 K^b^		777 K^b^	
			GEBV	DGV	GEBV	DGV	GEBV	DGV	GEBV	DGV
Traits in the selection index										
WGBW	0.64	0.64	0.67	0.42	0.67	0.42	0.67	0.43	0.66	0.42
WGWY	0.62	0.60	0.63	0.39	0.63	0.40	0.63	0.40	0.63	0.39
CW	0.60	0.61	0.64	0.40	0.64	0.40	0.64	0.40	0.63	0.39
CY	0.62	0.61	0.64	0.40	0.64	0.40	0.65	0.41	0.64	0.40
PW	0.60	0.61	0.64	0.40	0.64	0.40	0.64	0.40	0.63	0.39
PY	0.62	0.61	0.64	0.40	0.64	0.40	0.65	0.41	0.64	0.40
MW	0.60	0.61	0.64	0.40	0.64	0.40	0.64	0.40	0.63	0.39
MY	0.62	0.61	0.64	0.40	0.64	0.40	0.65	0.41	0.64	0.40
SCa	0.74	0.70	0.72	0.39	0.72	0.39	0.72	0.39	0.72	0.39
SCaw	0.73	0.70	0.71	0.38	0.71	0.39	0.71	0.39	0.71	0.38
	0.64	0.63	0.66	0.40	0.66	0.40	0.66	0.40	0.65	0.40
Traits not included in the selection index										
BW	0.66	0.65	0.68	0.44	0.68	0.44	0.68	0.44	0.68	0.43
BA	0.73	0.73	0.75	0.43	0.75	0.43	0.75	0.43	0.74	0.42
SW	0.60	0.61	0.63	0.40	0.64	0.40	0.64	0.40	0.63	0.39
SY	0.69	0.68	0.71	0.43	0.71	0.44	0.71	0.44	0.71	0.43
NW	0.72	0.71	0.74	0.47	0.74	0.47	0.74	0.47	0.74	0.46
NY	0.68	0.68	0.70	0.43	0.70	0.44	0.70	0.44	0.70	0.43
HW	0.58	0.55	0.58	0.35	0.58	0.35	0.58	0.35	0.58	0.35
HY	0.61	0.60	0.63	0.40	0.63	0.40	0.63	0.40	0.63	0.39
TR	0.52	0.51	0.53	0.31	0.53	0.31	0.53	0.31	0.53	0.31
OP	0.57	0.54	0.57	0.35	0.57	0.35	0.57	0.35	0.57	0.35
Average	0.64	0.63	0.65	0.40	0.65	0.40	0.65	0.40	0.65	0.40
Overall mean	0.64	0.63	0.65	0.40	0.65	0.40	0.66	0.40	0.65	0.39

^a^ Expected EBV, DGV and GEBV accuracy means were obtained from the mixed model equation; ^b^ 8 K: means that the base panel is the 8 K SNP panel imputed to the 50 K SNP panel; 15 K: means that the base panel is the 15 K SNP panel imputed to the 50 K SNP panel; 50 K: means that the true 50 K SNP panel; 777 K: means that the base panel is the 50 K SNP panel imputed to the 777 K SNP panel; ^c^ Abbreviation descriptions are presented in Table 1

## Discussion

Wide application of GS in beef cattle breeding programs depends among other factors, on the price of genotyping. The current medium or high density SNP chips are still expensive for widespread use in the beef industry, considering the number of individuals needed for reasonably accurate genomic predictions of breeding values. Genotype imputation has been used as an alternative to reduce costs [21, 23, 33, 34]. In this study, we investigated different scenarios as alternatives to use imputed genotypes in commercial beef cattle breeding programs. The correlation between DGV and EBV (validation accuracy) has been used to represent the accuracy of DGV [6, 35, 36]. The validation accuracy for traits in the selection index were lower than values reported in the literature for other breeds such as Angus, Limousin and Simmental [35, 36]. Neves et al. [32], working with Brazilian Nellore and the same set of traits (included in the selection index) also reported greater validation accuracies, except for WGBW and CW. The lower values of validation accuracy in this study could be explained by the lower expected EBV accuracies in the training population (*r* = 0.64). The greater validation accuracies observed for traits with higher heritability estimates (e.g., post weaning traits) has also been reported in the literature (e.g. Brito et al. [6] working with simulated data of beef cattle, Akanno et al. [37] studying pigs, and Khatkar et al. [33] working with Australian dairy cattle). In general, high heritability traits are associated with larger accuracy estimates. The reason why scrotal circumference (i.e. high heritability trait) presented the lowest validation accuracy could be due to the smaller number of animals in the training population (n = 708) compared to the other traits, as the size of training population is another very important component for the accuracy of genomic predictions.

The results from SCE1 and SCE2 showed that the size of the training population was more important than the percentage of animals with imputed genotypes. Other studies in dairy cattle have also reported small reduction in accuracies when using imputed genotypes to predict the effect of the markers [20, 21, 23, 33, 38]. These findings indicate that in order to improve the accuracies of genomic predictions in Brazilian Braford and Hereford, it is important to increase the size of the training population. It could be done by genotyping more animals with 50 K SNP chip panel or with 8 K or 15 K and impute to 50 K. On the basis of the relatively small reduction in accuracy of genomic prediction when using imputed genotypes, we would then recommend the use of 15 K for large-scale genotyping as long as its costs are acceptable to Brazilian Braford and Hereford breeders.

As discussed in Piccoli et al. [19] including pedigree information did not increase concordance rate or allelic R^2^. This could be expected due to the weak structure of the pedigree within the set of genotyped animals and in the whole pedigree file. Similar results were found by Carvalheiro et al. [22] when working with Nellore in Brazil with similar pedigree structure. It is also important to highlight that the dataset used for this investigation is from a commercial breeding program and not from research herds. Therefore, in practice there will be always an interest on predicting young animals based on the information from previous generations (with phenotypes and genotypes).

In theory, increasing the number of SNPs in a panel will increase the level of linkage disequilibrium (LD) between a SNP and a QTL and consequently there should be an improvement in accuracies of genomic predictions of breeding values. This assumption has been confirmed by other studies in the literature that reported increased prediction accuracies when using imputed HD genotypes compared to medium density genotypes (e.g. 50 K). For instance, Boison et al. [39], working with Guzerá (*Bos indicus*) cattle, reported an increase of 8% (averaged across all traits) when using imputed HD genotypes compared to true 50 K genotypes. Brito et al. [6], in a study with simulated data of beef cattle reported an increase of 0.09 in the DGV accuracy by using a 777 K SNP panel compared to a 50 K SNP panel. Weigel et al. [22] and Vazquez et al. [40] also reported higher accuracies of prediction using denser SNP markers. Other studies have reported gains in accuracy, even smaller, when using imputed 777 K compared to 50 K [34, 41, 42]. In the present study, we also investigated genotype imputation from 50 K to 777 K in Brazilian Braford and Hereford. However, no major advantages of the 777 K over the 50 K were observed. The losses in accuracies obtained for GEBV and DGV when using 777 K (SCE3) are probably associated with a higher percentage of imputation errors, as in this scenario only 212 animals had true HD genotypes [19]. Therefore, our results do not support the use of an imputed 777 K SNP chip panel to increase genomic prediction accuracies in Brazilian Braford and Hereford breeds. Similarly to our study, Su et al. [42] reported no gain in prediction accuracy when using imputed 777 K genotypes vs. the 50 K in Nordic Holstein and Red Dairy cattle.

Despite the losses in expected GEBV accuracies when using 8 K and 15 K SNP panels were statistically significant when compared to the ones attained using the true 50 K SNP chip panel, they were smaller with the 15 K compared to 8 K SNP chip panel. These results can be explained by the highest concordance rate in the 50 K SNP panel imputed from the 15 K SNP panel [19], lower genotyping errors and denser genome coverage. Similar trend was reported by Segelke et al. [38] when they analyzed the losses in reliability from imputed panels of two different densities of SNPs in German dairy cattle. Sargolzaei et al. [43], working with Canadian dairy cattle and 3 K SNP chip panel, also reported losses in reliability around −0.02. Boison et al. [39] also reported a small loss in accuracy of prediction using imputed 50 K from 3 K, while prediction accuracy remained the same for the imputed 50 K from 7 K. Chen et al. [44], working with Canadian dairy cattle, reported that the 6 K SNP (imputed to 50 K) panel performed better than the 3 K (imputed to 50 K) panel and resulted in the least reduction of genomic prediction accuracy among all the low-density panels evaluated in their study. The authors also reported that including genotypes imputed from the 6 K panel achieved almost the same accuracy of genomic prediction as that of using the 50 K panel even when 66% of the training population was genotyped on the 6 K panel. Our results and reports from the literature suggests that genomic prediction of breeding values derived from genotypes imputed from higher density SNP chip panels provide greater accuracies and in some cases, even the same than using the true 50 K genotypes. Similar trend was pointed out in a review study by Calus et al. [45], where the authors reported that in dairy cattle within-breed genomic predictions, the use of imputed 50 K genotypes typically yields 85% to almost 100% of the reliability obtained with a 50 K panel, provided that the low-density panel contains at least 3 K genotypes. Other studies in dairy cattle have shown that further imputation to 777 k SNPs yielded at most a limited further increase in reliability of genomic breeding values for within breed genomic prediction.

The traits related to fitness (NW, NY, HW, HY, TR and OP) had higher values of validation accuracy in relation to the other traits, including those from the selection index. These higher values are probably associated with greater genetic variability due to a weaker selection. The results found by Akanno et al. [37], working with simulated data in swine strengthens this theory because they found much higher accuracy for the indigenous population (low selection pressure) in comparison with the exotic population (high selection pressure). However, Neves et al. [32] studying Brazilian Nellore reported validation accuracy lower than those attained for the NW and NY traits in the current study. This could be due to a stronger selection pressure in Nellore breed compared to Braford and Hereford. Another explanation could be due to the fact that fitness traits in general have lower heritability estimates potentially due to the influence of non-additive genetic factors [46, 47]. The validation accuracy for the BW and the BA in this study were lower than other traits studied. This could be related to the strong selection, which is carried out in Hereford breed for these traits. Saatchi et al. [35, 48] working with Angus, Limousin, Simmental and Hereford breeds, found higher validation accuracy for these two traits compared to those reported in this study.

Validation accuracies for BW in the SCE2 scenario were not influenced by either the panel or the percentage of imputed animals in the training population. Different results were observed in SCE1, where both the panel and the number of animals in the training population influenced the validation accuracy of BW. Hayes et al. [11] showed that the values of genomic prediction accuracies are influenced by the size of the training population and Brito et al. [6] working with simulated data from beef cattle, also showed that the size of the training population has a major effect on the accuracies. Similar results were observed for the fitness traits (NW, NY, HW, HY, TR and OP). In general, the expected DGV accuracy for all traits across levels of each scenario were lower than the accuracy of the parents’ average as reported by Brito et al. [6] which were between 0.44 and 0.58 for traits with heritability estimates from 0.10 to 0.40.

The losses in expected GEBV accuracy in each scenario were always analyzed relatively to the scenario where only true genotypes were used. For traits not included in the selection index over the different scenarios, the losses in expected GEBV accuracy were, on average, higher compared to the group of traits included in the selection index used in the Brazilian Braford and Hereford breeding program. However, the losses in expected GEBV accuracy for the majority of traits were greater when using 50 K genotypes imputed from the 8 K SNP panel compared to 15 K. A greater percentage of animals with imputed genotypes was associated to higher losses in expected GEBV accuracy, regardless of the scenario investigated. In other words, losses in expected GEBV accuracy were higher due to greater error rates in the genotyping imputation process [19].

## Conclusions

The percentage of animals with imputed genotypes in the training population did not significantly influence the validation accuracy (Pearson’s correlation), but the size of the training population did influence the validation accuracies. The losses in expected GEBV accuracy due to imputation of genotypes were lower when using the 50 K SNP panel imputed from the 15 K SNP panel instead of imputation from the 8 K SNP panel. Therefore, using the low-density panels may allow Brazilian Braford and Hereford cattle breeders to genotype more animals, preferentially using 15 K or 50 K SNP chip panels, and consequently enlarging the size of the training population, which might in fact increase the accuracy of the DGV.

## Additional files

Additional file 1: Table S1. Losses in expected GEBV accuracy using the 8 K and 15 K SNP panel imputed to the 50 K SNP panel compared to the real 50 K SNP panel in the SCE1 scenarios. (DOC 61 kb)
Additional file 2: Table S2. Losses in expected GEBV accuracy using the 8 K and 15 K SNP panel imputed to the 50 K SNP panel in the SCE2 scenarios and the 777 K SNP panel imputed from the 50 K SNP panel in the SCE3 scenario compared to the true 50 K SNP panel. (DOC 63 kb)

## Abbreviations

BA: Birth assistance score
BW: Birth weight
CW: Conformation score at weaning
CY: Conformation score at yearling
DGV: Direct genomic breeding value
EBV: Estimated breeding value
GBLUP: Genomic Best Linear Unbiased Prediction
GEBV: Genomic estimated breeding value
GS: Genomic selection
HW: Hair length score at weaning
HY: Hair length score at yearling
MW: Muscularity score at weaning
MY: Muscularity score at yearling
NW: Prepuce (navel) score at weaning
NY: Prepuce (navel) score at yearling
OP: Ocular pigmentation score
PW: Precocity score at weaning
PY: Precocity score at yearling
QTL: Quantitative trait loci
SCa: Scrotal circumference adjusted for age at yearling
SCaw: Scrotal circumference adjusted for age and weight at yearling
SNP: Single Nucleotide Polymorphisms
SW: Size score at weaning
SY: Size score at yearling
TR: Tick resistance
WGBW: Weight gain from birth to weaning
WGWY: Weight gain from weaning to yearling
