# Supplementary material for: Genomic predictions for economically important traits in Brazilian Braford and Hereford beef cattle using true and imputed genotypes
Source: BMC Genet. 2017 Jan 18;18:2. doi: 10.1186/s12863-017-0475-9 (PMC5241971; doi:10.1186/s12863-017-0475-9)
Supplement: Additional file 1: Table S1. — Losses in expected GEBV accuracy using the 8 K and 15 K SNP panel imputed to the 50 K SNP panel compared to the real 50 K SNP panel in the SCE1 scenarios. (DOC 61 kb) [file 12863_2017_475_MOESM1_ESM.doc]

| **Table S1.** Losses in expected GEBV accuracy using the 8K and 15K SNP panel imputed to the 50K SNP panel compared to the real 50K SNP panel in the SCE1 scenarios^12^. | | | | | | | | | | | | |
| --- | --- | --- | --- | --- | --- | --- | --- | --- | --- | --- | --- | --- |
| **Traits^3^** | **8K^4^** | | | | | | **15K^4^** | | | | | |
|  | **10** | **20** | **30** | **40** | **50** | **60** | **10** | **20** | **30** | **40** | **50** | **60** |
| **WGBW** | -0.0008 | -0.0009 | -0.0005 | -0.0009 | -0.0009 | -0.0010 | -0.0003 | -0.0003 | 0.0001 | -0.0003 | -0.0004 | -0.0004 |
| **WGWY** | -0.0010 | -0.0011 | -0.0011 | -0.0011 | -0.0012 | -0.0008 | -0.0004 | -0.0004 | -0.0004 | -0.0004 | -0.0004 | 0.0000 |
| **CW** | -0.0010 | -0.0011 | -0.0012 | -0.0011 | -0.0011 | -0.0012 | -0.0004 | -0.0004 | -0.0004 | -0.0004 | -0.0004 | -0.0004 |
| **CY** | -0.0009 | -0.0010 | -0.0010 | -0.0011 | -0.0011 | -0.0012 | -0.0004 | -0.0004 | -0.0004 | -0.0004 | -0.0004 | -0.0004 |
| **PW** | -0.0010 | -0.0011 | -0.0012 | -0.0011 | -0.0011 | -0.0012 | -0.0004 | -0.0004 | -0.0004 | -0.0004 | -0.0004 | -0.0004 |
| **PY** | -0.0009 | -0.0010 | -0.0010 | -0.0011 | -0.0011 | -0.0012 | -0.0004 | -0.0004 | -0.0004 | -0.0004 | -0.0004 | -0.0004 |
| **MW** | -0.0010 | -0.0011 | -0.0012 | -0.0011 | -0.0011 | -0.0012 | -0.0004 | -0.0004 | -0.0004 | -0.0004 | -0.0004 | -0.0004 |
| **MY** | -0.0009 | -0.0010 | -0.0010 | -0.0011 | -0.0011 | -0.0012 | -0.0004 | -0.0004 | -0.0004 | -0.0004 | -0.0004 | -0.0004 |
| **SCa** | -0.0008 | -0.0009 | -0.0010 | -0.0010 | -0.0011 | -0.0012 | -0.0003 | -0.0004 | -0.0004 | -0.0004 | -0.0005 | -0.0005 |
| **SCaw** | -0.0008 | -0.0009 | -0.0010 | -0.0011 | -0.0011 | -0.0012 | -0.0003 | -0.0004 | -0.0004 | -0.0005 | -0.0005 | -0.0005 |
| **Average** | **-0.0009** | **-0.0010** | **-0.0010** | **-0.0011** | **-0.0011** | **-0.0011** | **-0.0004** | **-0.0004** | **-0.0004** | **-0.0004** | **-0.0004** | **-0.0004** |

^1^ Expected GEBV accuracy means that accuracies were obtained from the mixed model equation in the validation population; ^2^ SCE1 scenario that the number of animals and the percentage of animals with imputed genotypes in the training population varied; ^3^ WGBW: Weight gain from birth to weaning (kg); WGWY: Weight gain from weaning to yearling (kg); CW: Conformation score at weaning (scores 1-5); CY: Conformation score at yearling (scores 1-5); PW: Precocity score at weaning (scores 1-5); PY: Precocity score at yearling (scores 1-5); MW: Muscularity score at weaning (scores 1-5); MY: Muscularity score at yearling (scores 1-5); SCa: Scrotal circumference adjusted for age at yearling (cm); SCaw: Scrotal circumference adjusted for age and weight at yearling (cm); ^4^ 8K: means that the base panel is the 8K SNP panel imputed to the 50K SNP panel; 15K: means that the base panel is the 15K SNP panel imputed to the 50K SNP panel and 10, 20, 30, 40, 50 and 60 means the percentage of animals with imputed genotypes.

| **Table S1.** Cont. | | | | | | | | | | | | |
| --- | --- | --- | --- | --- | --- | --- | --- | --- | --- | --- | --- | --- |
| **Traits^3^** | **8K^4^** | | | | | | **15K^4^** | | | | | |
|  | **10** | **20** | **30** | **40** | **50** | **60** | **10** | **20** | **30** | **40** | **50** | **60** |
| **BW** | -0.0007 | -0.0008 | -0.0008 | -0.0008 | -0.0009 | -0.0009 | -0.0003 | -0.0003 | -0.0003 | -0.0003 | -0.0003 | -0.0003 |
| **BA** | -0.0004 | -0.0004 | -0.0005 | -0.0006 | -0.0006 | -0.0007 | -0.0002 | -0.0002 | -0.0002 | -0.0002 | -0.0003 | -0.0003 |
| **SW** | -0.0011 | -0.0011 | -0.0012 | -0.0011 | -0.0011 | -0.0012 | -0.0004 | -0.0004 | -0.0005 | -0.0004 | -0.0004 | -0.0004 |
| **SY** | -0.0007 | -0.0008 | -0.0009 | -0.0009 | -0.0010 | -0.0010 | -0.0003 | -0.0003 | -0.0003 | -0.0004 | -0.0004 | -0.0004 |
| **NW** | -0.0005 | -0.0006 | -0.0006 | -0.0007 | -0.0007 | -0.0008 | -0.0002 | -0.0002 | -0.0002 | -0.0003 | -0.0003 | -0.0003 |
| **NY** | -0.0007 | -0.0007 | -0.0008 | -0.0008 | -0.0009 | -0.0010 | -0.0003 | -0.0003 | -0.0003 | -0.0003 | -0.0004 | -0.0004 |
| **HW** | -0.0014 | -0.0015 | -0.0016 | -0.0017 | -0.0018 | -0.0018 | -0.0006 | -0.0006 | -0.0007 | -0.0007 | -0.0007 | -0.0008 |
| **HY** | -0.0009 | -0.0010 | -0.0011 | -0.0011 | -0.0012 | -0.0013 | -0.0004 | -0.0004 | -0.0004 | -0.0004 | -0.0005 | -0.0005 |
| **TR** | -0.0016 | -0.0017 | -0.0018 | -0.0019 | -0.0020 | -0.0020 | -0.0007 | -0.0007 | -0.0007 | -0.0008 | -0.0008 | -0.0008 |
| **OP** | -0.0015 | -0.0016 | -0.0017 | -0.0018 | -0.0019 | -0.0019 | -0.0006 | -0.0007 | -0.0007 | -0.0008 | -0.0008 | -0.0008 |
| **Average** | **-0.0010** | **-0.0010** | **-0.0011** | **-0.0012** | **-0.0012** | **-0.0013** | **-0.0004** | **-0.0004** | **-0.0004** | **-0.0005** | **-0.0005** | **-0.0005** |
| ^1^Expected GEBV accuracy means that accuracies were obtained from the mixed model equation in the validation population; ^2^ SCE1 scenario that the number of animals and the percentage of animals with imputed genotypes in the training population varied; ^3^BW: Birth weight (kg); BA: Birth assistance score (scores 1-5); SW: Size score at weaning (scores 1-5); SY: Size score at yearling (scores 1-5); NW: Prepuce (navel) score at weaning (scores 1-5); NY: Prepuce (navel) score at yearling (scores 1-5); HW: Hair length score at weaning (scores 1-3); HY: Hair length score at yearling (scores 1-3); TR: Ticks resistance (ticks unit); OP: Ocular pigmentation score (scores 1-3); ^4^ 8K: means that the base panel is the 8K SNP panel imputed to the 50K SNP panel; 15K: means that the base panel is the 15K SNP panel imputed to the 50K SNP panel and 10, 20, 30, 40, 50 and 60 means the percentage of animals with imputed genotypes. | | | | | | | | | | | | |
