# Supplementary material for: Genomic predictions for economically important traits in Brazilian Braford and Hereford beef cattle using true and imputed genotypes
Source: BMC Genet. 2017 Jan 18;18:2. doi: 10.1186/s12863-017-0475-9 (PMC5241971; doi:10.1186/s12863-017-0475-9)
Supplement: Additional file 2: Table S2. — Losses in expected GEBV accuracy using the 8 K and 15 K SNP panel imputed to the 50 K SNP panel in the SCE2 scenarios and the 777 K SNP panel imputed from the 50 K SNP panel in the SCE3 scenario compared to the true 50 K SNP panel. (DOC 63 kb) [file 12863_2017_475_MOESM2_ESM.doc]

| **Table S2.** Losses in expected GEBV accuracy using the 8K and 15K SNP panel imputed to the 50K SNP panel in the SCE2 scenarios and the 777K SNP panel imputed from the 50K SNP panel in the SCE3 scenario compared to the true 50K SNP panel^12^. | | | | | | | | | | | | | |
| --- | --- | --- | --- | --- | --- | --- | --- | --- | --- | --- | --- | --- | --- |
| **Traits^3^** | **8K^4^** | | | | | | **15K^4^** | | | | | | **777K^4^** |
|  | **10** | **20** | **30** | **40** | **50** | **60** | **10** | **20** | **30** | **40** | **50** | **60** |  |
| **WGBW** | -0.0001 | -0.0003 | -0.0004 | -0.0004 | -0.0006 | -0.0006 | 0.0002 | 0.0001 | 0.0001 | 0.0001 | 0.0000 | 0.0000 | -0.0020 |
| **WGWY** | -0.0002 | -0.0004 | -0.0005 | -0.0006 | -0.0008 | -0.0009 | 0.0002 | 0.0001 | 0.0001 | 0.0001 | 0.0000 | -0.0001 | -0.0020 |
| **CW** | -0.0008 | -0.0009 | -0.0010 | -0.0010 | -0.0011 | -0.0012 | -0.0003 | -0.0004 | -0.0004 | -0.0004 | -0.0004 | -0.0005 | -0.0022 |
| **CY** | -0.0007 | -0.0009 | -0.0009 | -0.0010 | -0.0012 | -0.0012 | -0.0002 | -0.0003 | -0.0004 | -0.0004 | -0.0005 | -0.0005 | -0.0026 |
| **PW** | -0.0008 | -0.0009 | -0.0010 | -0.0010 | -0.0011 | -0.0012 | -0.0003 | -0.0004 | -0.0004 | -0.0004 | -0.0004 | -0.0005 | -0.0023 |
| **PY** | -0.0007 | -0.0009 | -0.0009 | -0.0010 | -0.0012 | -0.0012 | -0.0002 | -0.0003 | -0.0004 | -0.0004 | -0.0005 | -0.0005 | -0.0026 |
| **MW** | -0.0008 | -0.0009 | -0.0010 | -0.0010 | -0.0011 | -0.0012 | -0.0003 | -0.0004 | -0.0004 | -0.0004 | -0.0004 | -0.0005 | -0.0023 |
| **MY** | -0.0007 | -0.0009 | -0.0009 | -0.0010 | -0.0012 | -0.0012 | -0.0002 | -0.0003 | -0.0004 | -0.0004 | -0.0005 | -0.0005 | -0.0026 |
| **SCa** | -0.0005 | -0.0006 | -0.0007 | -0.0008 | -0.0009 | -0.0011 | -0.0002 | -0.0002 | -0.0003 | -0.0003 | -0.0004 | -0.0005 | -0.0013 |
| **SCaw** | -0.0006 | -0.0006 | -0.0007 | -0.0008 | -0.0009 | -0.0011 | -0.0002 | -0.0002 | -0.0003 | -0.0003 | -0.0004 | -0.0005 | -0.0013 |
| **Average** | **-0.0006** | **-0.0007** | **-0.0008** | **-0.0009** | **-0.0010** | **-0.0011** | **-0.0002** | **-0.0002** | **-0.0003** | **-0.0003** | **-0.0003** | **-0.0004** | **-0.0021** |
| ^1^ Expected GEBV accuracy means that accuracy were obtained from the mixed model equation in the validation population; ^2^ SCE2 scenario that the percentage of animals with imputed genotypes in the training population varied and SCE3 scenario was created with only one percentage of animals with imputed genotypes and only one training population size; ^3^ WGBW: Weight gain from birth to weaning (kg); WGWY: Weight gain from weaning to yearling (kg); CW: Conformation score at weaning (scores 1-5); CY: Conformation score at yearling (scores 1-5); PW: Precocity score at weaning (scores 1-5); PY: Precocity score at yearling (scores 1-5); MW: Muscularity score at weaning (scores 1-5); MY: Muscularity score at yearling (scores 1-5); SCa: Scrotal circumference adjusted for age at yearling (cm); SCaw: Scrotal circumference adjusted for age and weight at yearling (cm); ^4^ 8K: means that the base panel is the 8K SNP panel imputed to the 50K SNP panel; 15K: means that the base panel is the 15K SNP panel imputed to the 50K SNP panel; 777K: means that the base panel is the 50K SNP panel imputed to the 777K SNP panel and 10, 20, 30, 40, 50 and 60 means the percentage of animals with imputed genotypes. | | | | | | | | | | | | | |

| **Table S2.** Cont. | | | | | | | | | | | | | |
| --- | --- | --- | --- | --- | --- | --- | --- | --- | --- | --- | --- | --- | --- |
| **Traits^3^** | **8K^4^** | | | | | | **15K^4^** | | | | | | **777K^4^** |
|  | **10** | **20** | **30** | **40** | **50** | **60** | **10** | **20** | **30** | **40** | **50** | **60** |  |
| **BW** | -0.0005 | -0.0007 | -0.0007 | -0.0008 | -0.0009 | -0.0010 | -0.0002 | -0.0003 | -0.0003 | -0.0003 | -0.0004 | -0.0004 | -0.0021 |
| **BA** | -0.0004 | -0.0003 | -0.0004 | -0.0004 | -0.0005 | -0.0006 | -0.0002 | -0.0001 | -0.0002 | -0.0002 | -0.0002 | -0.0003 | -0.0020 |
| **SW** | -0.0008 | -0.0010 | -0.0010 | -0.0010 | -0.0012 | -0.0012 | -0.0003 | -0.0004 | -0.0004 | -0.0004 | -0.0004 | -0.0005 | -0.0022 |
| **SY** | -0.0005 | -0.0006 | -0.0007 | -0.0008 | -0.0009 | -0.0011 | -0.0002 | -0.0002 | -0.0003 | -0.0003 | -0.0004 | -0.0005 | -0.0024 |
| **NW** | -0.0004 | -0.0006 | -0.0006 | -0.0007 | -0.0008 | -0.0008 | -0.0002 | -0.0002 | -0.0002 | -0.0003 | -0.0003 | -0.0003 | -0.0024 |
| **NY** | -0.0005 | -0.0007 | -0.0007 | -0.0008 | -0.0010 | -0.0010 | -0.0002 | -0.0003 | -0.0003 | -0.0003 | -0.0004 | -0.0004 | -0.0025 |
| **HW** | -0.0015 | -0.0015 | -0.0016 | -0.0016 | -0.0018 | -0.0020 | -0.0006 | -0.0006 | -0.0007 | -0.0007 | -0.0008 | -0.0009 | -0.0014 |
| **HY** | -0.0007 | -0.0009 | -0.0010 | -0.0010 | -0.0012 | -0.0013 | -0.0003 | -0.0004 | -0.0004 | -0.0004 | -0.0005 | -0.0005 | -0.0027 |
| **TR** | -0.0016 | -0.0016 | -0.0017 | -0.0017 | -0.0018 | -0.0020 | -0.0006 | -0.0007 | -0.0007 | -0.0007 | -0.0008 | -0.0009 | -0.0016 |
| **OP** | -0.0017 | -0.0017 | -0.0019 | -0.0019 | -0.0020 | -0.0022 | -0.0007 | -0.0007 | -0.0008 | -0.0008 | -0.0008 | -0.0010 | -0.0011 |
| **Average** | **-0.0009** | **-0.0010** | **-0.0010** | **-0.0011** | **-0.0012** | **-0.0013** | **-0.0003** | **-0.0004** | **-0.0004** | **-0.0004** | **-0.0005** | **-0.0006** | **-0.0021** |
| ^1^ Expected GEBV accuracy means that accuracy were obtained from the mixed model equation in the validation population; ^2^ SCE2 scenario that the percentage of animals with imputed genotypes in the training population varied and SCE3 scenario was created with only one percentage of animals with imputed genotypes and only one training population size; ^3^BW: Birth weight (kg); BA: Birth assistance score (scores 1-5); SW: Size score at weaning (scores 1-5); SY: Size score at yearling (scores 1-5); NW: Prepuce (navel) score at weaning (scores 1-5); NY: Prepuce (navel) score at yearling (scores 1-5); HW: Hair length score at weaning (scores 1-3); HY: Hair length score at yearling (scores 1-3); TR: Ticks resistance (ticks unit); OP: Ocular pigmentation score (scores 1-3); ^4^8K: means that the base panel is the 8K SNP panel imputed to the 50K SNP panel; 15K: means that the base panel is the 15K SNP panel imputed to the 50K SNP panel; 777K: means that the base panel is the 50K SNP panel imputed to the 777K SNP panel and 10, 20, 30, 40, 50 and 60 means the percentage of animals with imputed genotypes. | | | | | | | | | | | | | |
